# Positionspapier Schlaganfallnachsorge der Deutschen Schlaganfall-Gesellschaft – Teil 1: Nachsorge nach einem Schlaganfall: Status quo der Versorgungsrealität und Versorgungsdefizite in Deutschland

**DOI:** 10.1007/s00115-021-01231-9

**Published:** 2022-01-03

**Authors:** Stephen Kaendler, Martin Ritter, Dirk Sander, Matthias Elstner, Christopher Schwarzbach, Markus Wagner, Andreas Meisel, Mitglieder Kommission Nachsorge der Deutschen Schlaganfall-Gesellschaft

**Affiliations:** 1 Praxis Kaendler & Wurtz, Praxis für Nervenheilkunde, Kaiserstraße 75, 63065 Offenbach, Deutschland; 2 Praxis Böckenholt & Ritter, Münster, Deutschland; 3 Neurozentrum Tutzing-Feldafing, Benedictus-Krankenhaus, Tutzing, Deutschland; 4 Klinik für Neurologie, Klinikum Ansbach, Ansbach, Deutschland; 5 TUM, München, Deutschland; 6 Klinik für Neurologie, Klinikum Ludwigshafen, Ludwigshafen, Deutschland; 7 Stiftung Deutsche Schlaganfall-Hilfe, Gütersloh, Deutschland; 8 Centrum für Schlaganfallforschung Berlin und Klinik und Hochschulambulanz für Neurologie, Charité – Universitätsmedizin Berlin, corporate member of Freie Universität Berlin, Humboldt-Universität zu Berlin, and Berlin Institute of Health (BIH), Berlin, Deutschland

**Keywords:** Schlaganfall, Ambulante Nachsorge, Langzeitkonzept, Unberücksichtigte Domänen, Rehabilitation, Stroke, Outpatient aftercare, Long-term management, Unmet domains, Rehabilitation

## Abstract

Die Akutversorgung des Schlaganfalls in Deutschland hat ein sehr hohes Niveau, dargestellt durch die Stroke-Units. Die Erkrankung Schlaganfall hat eine Akutphase, gefolgt von einer chronischen Phase mit einem hohen und qualifizierten multi- und interprofessionellen Versorgungsbedarf. Die Deutsche Schlaganfall-Gesellschaft (DSG) hat 2020 eine Nachsorgekommission gegründet, mit dem Ziel der Darstellung der aktuellen Versorgungssituation und zur Erarbeitung von Vorschlägen für eine Verbesserung der Versorgung nach der Akutphase. In dieser Arbeit wird der Status quo ermittelt und Defizite benannt. Analysiert wurden Beiträge unterschiedlicher Beteiligter im deutschen Gesundheitswesen, dargestellt werden unterschiedliche Projekte einer Nachsorge. In Deutschland existiert kein anerkanntes strukturiertes Nachsorgekonzept für Patienten nach einem Schlaganfall. Die bestehende hausarztbasierte Versorgung ohne eine zukünftig stärkere und abgestimmte Integration der Neurologen erschwert eine leitlinien- und qualitätsgesteuerte Nachsorge. Aufgabenverteilungen sowie notwendige Ausbildungsstandards für ihre leitliniengerechte Erfüllung durch die Fachgruppen liegen nicht vor. Zu selten werden neben den medizinischen Domänen die physischen, sozialen und emotionalen Domänen durch ein multiprofessionelles Versorgungsteam beachtet. Zu diskutieren ist eine Weiterentwicklung eines regionalen Care-Management-Konzeptes. Evaluiert werden müssen die Ergebnisse und die Kosten eines Nachsorgekonzeptes vor einer breiten Anwendung.

## Abstract (en)

The acute treatment of stroke patients in Germany is of a very high standard, guaranteed by its system of stroke units. Stroke as a disease has an acute phase followed by a chronic phase that requires a high level of qualified aftercare given by multidisciplinary and interdisciplinary teams. In 2020, the German Stroke Society (DSG) founded a commission for long-term stroke care. The aim is to evaluate the current situation of long-term aftercare and suggest improvements for its structure. In this paper the status quo of aftercare is presented and possible deficits are identified. Contributions of various stakeholders from the German healthcare system are analyzed and different projects for post-acute care are presented. Germany has no acknowledged structured aftercare concepts for patients after stroke. The general practitioner-based care is currently the focus of patient management but without a greater, more coordinated integration of neurologists, guideline-led and quality-controlled aftercare will be harder to implement in the future. The assignment of duties and the necessary training standards for the specialist groups in order to comply with the guidelines do not exist. Besides medical health, the needs of physical, social and emotional domains are too seldom considered by a multiprofessional care team. Further developments of a regional care management concept are discussed. The results and costs of any aftercare concepts must be evaluated before widespread implementation.

## Einleitung

„Trotz zunehmender Erfolge in der Akutbehandlung des Schlaganfalls zählt dieser nach wie vor zu den häufigsten Ursachen für erworbene Behinderung und Tod.“ Diese allgemeine Aussage findet sich in den Einleitungen fast aller Artikel zum Thema Schlaganfall. Die Mehrheit der Arbeiten beschäftigte sich fast ausschließlich mit der Akutversorgung der Patienten. Europaweit sind die Thrombolyse- und Thrombektomiezahlen in Deutschland beispielgebend. Durch die moderne „Schlaganfallmedizin“ wurde die Schlaganfallmortalität seit 1980 halbiert. Der primäre und wesentliche Grund dieser positiven Entwicklung ist die Behandlung von Schlaganfallpatienten auf spezialisierten Stroke-Units.

Die Deutsche Schlaganfall-Gesellschaft (DSG) hat 2020 eine Nachsorgekommission gegründet, um die Schlaganfallnachsorge konzeptionell weiterzuentwickeln. In der Kommission sollen die aktuelle Versorgungssituation dargestellt und Vorschläge für eine Verbesserung der Versorgung nach der Akutphase erarbeitet werden. Die Kommission soll sukzessive Schwachstellen und Optimierungsansätze aufdecken, besser operationalisieren und in der Folge tragfähige, möglichst evidenzbasierte Konzepte zur Nachsorge entwickeln. Die Akutbehandlung des Schlaganfalls ist fest in neurologischer Hand, im ICD 11 wird der Schlaganfall endgültig eine „Neurologische Erkrankung“ sein, daher ist eine umfassende Beschäftigung aller Neurologen mit diesem Krankheitsbild notwendig.

Der Schlaganfall ist eine langfristige Erkrankung mit einer Akutphase und einer nachfolgenden chronischen Phase mit sich draus ableitenden unterschiedlichen Behandlungsschwerpunkten [25]. Neben der Akutversorgung und der Rehabilitation gehören dazu auch die Erfassung und Behandlung der Risikofaktoren im ambulanten Bereich sowie die kompetente multiprofessionelle Versorgung der betroffenen Patienten unter Einbezug der Angehörigen (Tab. 1).

**Table 1:** 

Phase	Behandlungsschwerpunkt
1. Phase – akut	Behandlung des Akutereignisses auf der Stroke-Unit
2. Phase – subakut	Einleitung der Reha stationär/ambulant
3. Phase – subakut	Übergang in die ambulante Phase
4. Phase – chronisch	Ambulante Versorgung

Viele Patienten verlassen die Rehabilitation [13, 30] mit relevanten körperlichen und psychologischen Defiziten. Aus gesellschaftlicher Sicht haben Aphasien, kognitive Defizite und affektive Störungen einen großen Einfluss auf die Pflegebedürftigkeit und den langfristigen oder gar dauerhaften Bedarf an adäquater ambulanter medizinischer Versorgung. Frühzeitige soziale Angebote durch Sozialarbeiter verbessern und erweitern die weitere Behandlung und fördern die Reintegration [22]. Aufgrund der wirtschaftlichen und sozialen Bedeutung des Schlaganfalls ist ein standardisiertes Nachsorgekonzept, wie es in anderen Ländern [21] schon entwickelt wurde, auch für Deutschland notwendig.

## Status quo und Defizite in der Versorgungsstruktur der Schlaganfallerkrankung

Schon seit Jahrzehnten ist bekannt, dass die Nachsorge ein zentraler Aspekt der Versorgung von Schlaganfallpatienten ist:

In der Helsingborg Declaration, einem paneuropäischen Konsensus zum Thema Schlaganfall, wurden 1995 fünf Aspekte der Schlaganfallbehandlung aufgeführt [1]:

- Akutbehandlung des Schlaganfalls
- Rehabilitation
- Sekundärprävention
- Evaluation der Behandlungsergebnisse
- Organisation der Schlaganfallbehandlung

Essenziell wurde die Kontinuität nach der Entlassung aus dem Krankenhaus in die Rehabilitation und ambulante Versorgung dargestellt. In der nächsten Konferenz 2006 [15] wurden Ziele für 2015 formuliert: Drei Monate nach einem Schlaganfall sollten über 70 % der überlebenden Patienten unabhängig in den Aktivitäten des täglichen Lebens (ADL) sein. Die Patienten sollten in einer Versorgungskette aufgenommen werden, mit definierten erreichbaren Zielen in den personellen und sozialen Domänen.

Im Jahr 2018 wurde von der „European Stroke Organisation“ (ESO; [18]) zusammen mit der Patientenorganisation „Stroke Alliance for Europe“ (SAFE) ergänzend und erweiternd der „Stroke Action Plan for Europe“ (2018–2030) entwickelt. Dieser Aktionsplan definiert evidenzbasiert Ziele, die mit Unterstützung der jeweiligen Gesundheitspolitik bis 2030 in allen europäischen Ländern für jeden Patienten, unabhängig vom Wohnort oder Sozialstatus umgesetzt werden sollen. Der Aktionsplan beschreibt als Einzelziel für ganz Europa nationale Versorgungspläne, die die gesamte Versorgungskette von der Primärprävention bis zur Nachsorge umfassen. Die Schlaganfallnachsorge wird erstmalig in einer eigenständigen Domäne „Life After Stroke“ erwähnt und erste Key-Performance-Indikatoren benannt.

Die Analyse der tatsächlichen Verhältnisse durch verschiedene Institutionen und Kostenträger hat erhebliche Defizite aufgezeigt, die bis heute nicht adäquat behoben sind:

Im Jahr 2001 kam der *Sachverständigenrat für die Konzertierte Aktion im Gesundheitswesen* (SVR‑G 2001) zu dem Schluss, dass trotz zahlreicher Bemühungen zur Verbesserung der Versorgungsqualität z. B. durch den Ausbau von Stroke-Units Defizite in der Patientenversorgung und speziell in der strukturierten Nachsorge verbleiben. Die Schnittstellen zwischen den Leistungssektoren sind als zentrale Schwachstellen des deutschen Gesundheitssystems bezeichnet worden.

- Der Sachverständigenrat präzisierte in seinem Gutachten von 2007 [24] mit dem Titel „Kooperation und Verantwortung: Voraussetzungen einer zielorientierten Gesundheitsversorgung“: Es braucht *ambulante multiprofessionelle Teams*, die die Versorgung einer älter werdenden, an chronischen und multiplen Erkrankungen leidenden Bevölkerung zur Aufgabe haben und alle Berufsgruppen umfassen, die für die Versorgung „in der Fläche“ notwendig sind.
- Ein *transsektorales „Case-Management“* ist notwendig, das die Fallführung in den drei Sektoren koordiniert und das die neuen Funktionen, vor allem hinsichtlich der patientenorientierten Abstimmung der Behandlung beim Übergang zwischen den Sektoren, und das Erreichen eines gemeinsamen Behandlungserfolges in den Mittelpunkt stellt.

Das *aQua Institut *[5] veröffentlichte 2015 eine Analyse im Auftrag des Gemeinsamen Bundesausschusses (G-BA) über die Versorgungsqualität bei Schlaganfall als Konzeptskizze für ein Qualitätssicherungsverfahren. Im stationären Bereich und in der Rehabilitation sei eine sektorenübergreifende Qualitätssicherung möglich.

Als erhebliche Mängel wurden aufgezeigt:

- Unzureichende Rehabilitation. Nur 41 % der Patienten erhielten eine neurologische Rehabilitation der Phase B bis D, 11 % eine geriatrische und 2 % erhielten sonstige Rehabilitationsmaßnahmen.
- Versorgung mit Logopädie und neuropsychologische Therapiemaßnahmen wurden kaum untersucht.
- Nachsorgeprogramme fehlten, qualitativ hochwertige Leitlinien und strukturierte Behandlungspfade in der Nachsorge ebenso wie „Disease-Management“-Programme.

Eine aktuelle Befragung von versicherten Patienten und Ärzten durch die Barmer Ersatzkasse [11] zeigte deutlich die nicht optimale Regelung der Schnittstellen zwischen stationärer Behandlung und ambulanter Versorgung. Insbesondere spielen niedergelassene Neurologen bei der Versorgung kaum eine Rolle. Als Folge kommt es zur Verzögerung der ambulanten rehabilitativen Maßnahmen:

- keine eindeutigen Aufgreifkriterien für Leistungen bei ambulanten Patienten mit funktionellem Defizit nach Schlaganfall,
- fehlende Standards der Qualitätssicherung zur Weiterführung der Sekundärprophylaxen, aufgezeigt am Beispiel der Fortführung der Verordnung von Thrombozytenaggregationshemmern und oralen Antikoagulanzien,
- Hinweise auf eine Unterversorgung von Depressionen mit antidepressiver Medikation und Psychotherapie im niedergelassenen Bereich,
- unzureichende Information von Patienten und deren Angehörigen.

### Nachsorgeprogramme und -projekte

Verschiedene Modelle der Nachsorge wurden in Deutschland beschrieben und evaluiert. Ziele der Studien waren die Überprüfung der Ergebnisqualität der akuten Schlaganfallversorgung und des Verlaufs [4]. Es wurden unterschiedlichste Methoden angewandt (Tab. 2).

**Table 2:** 

Name des Projektes	Kurzbeschreibung
Strukturierte ambulante Nachsorge nach Schlaganfall (SANO)	Cluster-randomisierte Studie Januar 2019 bis Juli 2021 zur Verbesserung der Versorgung nach einem ischämischen Schlaganfall. Entwicklung eines multidisziplinären Netzwerks und eine individuelle patientenzentrierte Intervention in Deutschland. Innovationsfondsförderung. Bisher keine Ergebnisse
INSPiRE-TMS	Offene, randomisiert kontrollierte Studie August 2011 bis Oktober 2017. Untersuchung eines Unterstützungsprogramms für eine erweiterte Sekundärprävention in Deutschland und Dänemark. Intensiviertes Unterstützungsprogramm ist mit einer höheren Zielerreichung in der Sekundärprävention verbunden. Keine signifikante Verringerung der Rate schwerwiegender vaskulärer Ereignisse
Stroke-Card	Randomisiert-kontrollierte Studie Januar 2014 bis Dezember 2017 zur Untersuchung der STROKE-CARD, einem Disease-Management-Programm bei Schlaganfallbetroffenen in Österreich. Die Versorgung STROKE-CARD reduziert die kumulierte einjährige Inzidenz kardiovaskulärer Erkrankungen, Verbesserung der Lebensqualität
Managing Aftercare for Stroke (MAS)	Beobachtungsstudie (MAS I) und anschließende Interventionsstudie (MAS II; April 2017 bis März 2021) in Berlin. Entwicklung eines integrierten Nachsorgemodells für Schlaganfallpatienten über die Phase der Rehabilitation hinaus. Patienten wurden nach der Entlassung in die Klinik eingeladen. Dort erfolgt eine ärztliche Untersuchung sowie ein Screening für offene medizinische/soziale Bedürfnisse, Nutzung von Scores für die Bestimmung des langfristigen Outcomes. Bisher keine Ergebnisse
SOS-Care – Hilfe nach Schlaganfall	Implementierung eines Schlaganfall-Lotsen am Universitätsklinikum Dresden (Selektivvertrag mit AOK PLUS). Fünfjährige Erprobungsphase ab 2009 und erste Studien zur Wirksamkeit des Nachsorgeprojekts. Weiterfinanzierung durch die AOK PLUS
Ergebnisqualität durch Patient Reported Outcome Measures (PROMs) bei Schlaganfallpatienten in der klinischen Routine – EPOS	Explorative Studie Januar 2017 bis Dezember 2020 zur Implementierung und Evaluation einer standardisierten Ergebnisqualitätsmessung unter Einbezug von Patient Reported Outcome Measures (PROMs) in die Routineversorgung von Schlaganfallpatienten im Universitätsklinikum Hamburg-Eppendorf (UKE). Nachverfolgung der Patienten bis zu einem Jahr nach Akutbehandlung. Innovationsfonds gefördert. Bisher keine Ergebnisse
StroCare – Optimierte sektorenübergreifende, koordinierte und evidenzbasierte Behandlung von Schlaganfallpatienten durch übergreifende Prozessverantwortung und patientenorientierte Ergebnisqualitätsmessung	Interventionsstudie von Juli 2019 bis Juni 2023 zur Untersuchung einer vernetzten Schlaganfallnachsorge und einer patientenzentrierten Messung der Ergebnisqualität am Universitätsklinikum Hamburg-Eppendorf (UKE). Innovationsfonds gefördert. Bisher keine Ergebnisse
STROKE-OWL	Interventionsstudie Oktober 2017 bis September 2021 zur Untersuchung von Schlaganfall-Lotsen in Ostwestfalen-Lippe (OWL) auf Basis von Primär- und Sekundärdaten. Innovationsfondsförderung und Überbrückungsfinanzierung bis Ergebnisse vorliegen
QUASCH – Ergebnisse qualitätsgesicherter Schlaganfallversorgung: Hessen im Vergleich zum übrigen Bundesgebiet	Effektivitätsuntersuchung der externen Qualitätssicherung beim akuten Schlaganfall auf Basis der Daten des Wissenschaftlichen Instituts der AOK (WidO) und der Geschäftsstelle Qualitätssicherung Hessen (GQH) 2007 bis 2017. Das Komplikationsrisiko aller Patienten sank im Vergleich der beiden Beobachtungszeiträume (2007–2010; 2014–2017) in Hessen signifikant
PostStroke-Manager	Machbarkeitsstudie des PostStroke-Manager-Systems 2021 in Leipzig. Untersuchung des hybriden Unterstützungskonzeptes bestehend aus einer Lotsenbetreuung und digitalen Tools (Nachsorge-App für Patienten, Web-Portal für Lotsen). Bisher keine Ergebnisse
INVADE Interventionsprojekt zerebrovaskuläre Erkrankungen und Demenzen im Landkreis Ebersberg	Interventionsstudie 2013 bis 2016 zur Untersuchung eines hausarztbasierten Präventionsprogramms bei zerebrovaskulären Erkrankungen und Demenzen. Die Ergebnisse stützen die Annahme, dass durch eine intensivierte hausärztliche Identifikation und Behandlung vaskulärer Risikofaktoren das Auftreten von zerebrovaskulären Erkrankungen und Demenzen gesenkt werden kann

Die Aufstellung ist nicht umfassend, es gibt z. B. weitere Projekte, die einen Case- und Care-Management-Ansatz verfolgen. Hierzu zählen das Projekt „Stroke Nurse“ in Ravensburg (www.strokenurse.de) oder das Projekt HANNS in Hanau. Ferner wurden am Klinikum Vest in Recklinghausen (Knappschaft) in Anlehnung an STROKE OWL mehrere Schlaganfall-Lotsen implementiert.

Viele Studien berücksichtigten neurologische Aspekte der Nachsorge bei der Studienkonzeption nicht. Zu nennen sind die neurologischen Folgen mit Schmerz, Spastik, Aphasie, Gangstörungen, Epilepsie, Depression, neurokognitive Defizite [7]. Hauptsächlich berücksichtigt werden die Punkte der Sekundärprävention. Die Autoren dieser Arbeit initiierten eine Umfrage (Zusammenarbeit der DSG, der Stiftung Schlaganfallhilfe und des Berufsverbandes BVDN) unter niedergelassenen Neurologen in Hessen zur Nachsorgesituation aus ambulanter neurologischer Perspektive. Die Auswertung ist noch nicht abgeschlossen und wird demnächst publiziert.

### Status quo

In der Regel steht bisher der Hausarzt [13] im Zentrum der Nachsorge. „Hausarztmodelle“ unterstützen dieses Konzept durch finanzielle Vergünstigungen für Patienten. Hausärzte agieren als Manager und vermitteln die notwendigen Therapien durch andere Versorger bzw. übernehmen einen Teil dieser Aufgaben selbst. Dieses Modell erlaubt zwar ein hohes Maß an Flexibilität, es entsteht aber eine ausgeprägte Variabilität hinsichtlich des Zugangs für Patienten zur qualitativ hochwertigen Nachsorge, einschließlich des Zugangs zu Hilfs- und Heilmitteln. Dieses Modell erschwert eine leitlinien- und qualitätsgesteuerte nachvollziehbare Behandlung. Eine regelhafte Einbindung von Schlaganfallspezialisten (Neurologen) in die Nachsorge ist nicht vorgesehen und erfolgt unstrukturiert.

Im Gegensatz zur Akutversorgung und frühen Rehabilitation existieren keine Leitlinien und keine Qualitätsindikatoren zur Messung der Behandlungsgüte oder interdisziplinäre „Comprehensive-care“-Ansätze. Es bieten sich zwar einige Strukturen an, innerhalb derer die Nachsorge verbessert werden könnte, die Konzepte stoßen auf die vielfach beschriebene Problematik der Sektorentrennung im deutschen Gesundheitssystem:

#### Regionale Versorgung.

Projekte zur integrierten Versorgung bedienen sich in unterschiedlicher Form und Intensität der in der Fachliteratur beschriebenen Elemente von „managed care“ mit sektorenübergreifender Koordination und Kooperation zwischen allen am Behandlungsablauf Beteiligten [6]. Managed care umfasst neben indikationsübergreifenden, populationsbezogenen Versorgungsnetzen u. a. Disease-Management und Case-Management. Disease-Management bezieht sich auf Patientengruppen mit chronischen Krankheiten. Case-Management ist konzentriert auf einzelne, zumeist kostenintensive Krankheitsfälle. Gemeinsam ist beiden Formen eine zielorientierte, sektorenübergreifende Koordination und Kooperation zwischen allen Beteiligten [24].

Die Stiftung Deutsche Schlaganfall-Hilfe führt in diesem Zusammenhang seit 2017 ein befristetes (4 Jahre) Projekt mit dem Namen STROKE OWL in mehreren Regionen durch [27]. Ziel ist es, die Versorgung von Schlaganfallpatienten durch eine flächendeckende Implementierung und Evaluation eines sektorenübergreifenden Versorgungsmanagements mit Lotsen nach erfolgtem Schlaganfall zu verbessern. Realistisch kann sich ein Lotse um maximal 100 Patienten kümmern. Sollten nur 20 % der Patienten mit einem Schlaganfall in Deutschland nach diesem Modell betreut werden, würden für die Umsetzung ca. 540 Lotsen benötigt. Denkbar ist die Weiterentwicklung und Umsetzung des Prinzips des Care-Managements als regionale Vernetzung, ausgehend und gesteuert von den Neurologen der Stroke-Units, wie dies im von der DSG initiierten SANO-Projekt erfolgt [9]. Evaluationsergebnisse beider im Rahmen des Innovationsfonds vom G‑BA geförderten Projekte (ab Ende 2021) inklusive der bedeutsamen gesundheitsökonomischen Analysen sind abzuwarten [10].

### Zwischenfazit und Empfehlung

- Alle in Tab. 2 dargestellten Modelle liefern Ansatzpunkte für eine Verbesserung der Nachsorge, wobei die nötige Evidenz zur Umsetzung auf Populationsebene fehlt.
- Ein „One-size-fits-it-all“-Ansatz wird von der Nachsorgekommission als unrealistisch angesehen. Strukturen in städtischen Bereichen und ländlichen Regionen sind unterschiedlich. Regionale Konzepte bieten den Vorteil, flexibel auf vorhandene Strukturen und Versorgungspfade zurückgreifen bzw. diese kooperativ weitergestalten zu können.
- Die Nachsorgekommission empfiehlt in diesem Zusammenhang, dass die DSG und Schlaganfall-Stiftung sich dieser Thematik der Verbesserung der sektoralen Vernetzung verstärkt annimmt und dabei auch die Entwicklung und Etablierung von Versorgungsstandards im ambulanten Bereich (Struktur- und Prozessqualität) vorantreibt.

## Weiterentwicklungsmöglichkeiten

### Qualitätssicherung

Betrachtet man die Inhalte und Ziele der Studien über die Nachsorge von Schlaganfallpatienten wird als messbarer Qualitätsindikator z. B. die Einstellung der Risikofaktoren bewertet. Daten von Nachuntersuchungen der Sekundärprävention [12] weisen auf ein Verbesserungspotenzial der Therapieadhärenz hin. Die Durchführung und das Ergebnis von Rehabilitationsverfahren (Physio‑, Ergo‑, Logopädie oder Neuropsychologie) zur Behandlung der neurologischen Defizite wird hingegen kaum betrachtet [31]. In der Erfahrung der Kommissionsmitglieder sind dies aber die Bereiche, welche wesentlich dazu beitragen, dass der Patient in sein tägliches Umfeld reintegriert wird [22]. Hier sollten dringend Konzepte zur Qualitätssicherung entwickelt werden.

### Checklisten und Assessments

In diesem Zusammenhang fällt auch auf, dass es keine definierten „Checklisten“ für die Nachsorge gibt, anhand derer man sich um den Patienten kümmern könnte, vorstellbar sind Aufgabenverteilungen in der Nachbetreuung. Modelle für Checklisten gibt es, zu erwähnen sind die Post Stroke Check List der WSO [14] und die Listen der Great Machchester Study. Bei der Erstellung einer Checkliste ist zu beachten, dass die verschiedenen Dimensionen möglichst umfänglich einbezogen werden. Die Liste darf aber nicht zu komplex (Zeit- und Personalaufwand) werden, damit es eine Chance auf Akzeptanz in der Praxis außerhalb von Studien gibt.

### Informationsfluss

Wichtig erscheint ein aktueller Informationsfluss über und zu den Patienten. Dieses setzt den zeitnahen Versand der Entlassberichte der akut versorgenden Stroke-Units an die Rehakliniken und die weiterbetreuenden Ärzte voraus. Der Entlassbrief soll eine konkrete Hilfestellung für die Beratung und Betreuung der Schlaganfallpatienten und deren Angehöriger geben. Diese Aufgabe könnte zu einem Element der Qualitätssicherung der Stroke-Units erweitert werden. Aus Sicht der Mitglieder wäre dieses ein Ansatz für eine Auflösung der Schnittstellenproblematik (Tab. 3).

**Table 3:** 

	Anforderung
1.	Durchgeführte Behandlung
2.	Befunde (neurologische Befunde, technische Untersuchungen)
3.	Ätiologische Zuordnung der Infarkte: TOAST-Klassifikation o. Ä., Blutungen: lobär/„loco typico“/andere Ursache
4.	Darstellung der Ausfälle des Patienten für eine Bewertung der Entwicklung im Verlauf (Modified Rankin Scale [mRS], NIHS)
5.	Begründung der vorgeschlagenen Medikation, Angabe von Zielwerten
6.	Empfehlungen zu Art und Zeit von Verlaufsuntersuchungen, Hinweise auf zu veranlassende Untersuchungen
7.	Rezidivrisiko in %/Jahr, erwarteter Nutzen bez. Rezidivrisiko
8.	Weitere Therapieempfehlung (Reha/ambulant oder stationär)
9.	Empfehlungen zu der Fahreignung
10.^a^	Stellungnahme zu einer Arbeitsmarktprognose
11.^a^	Empfehlungen zur Beantragung von Hilfen: – Versorgungsamt (Schwerbehindertenaus, Vergünstigungen) – Pflegegrad, ambulante Versorgungsnotwendigkeit durch Sozialstation – Einrichtung von Betreuung/Vorsorgevollmachten
12.^a^	Empfehlung zur Versorgung mit Hilfsmitteln
13.^a^	Empfehlungen zur Wohnsituation

^a^Entlassbeurteilung der Rehaklinik

### Neurologische Komorbidität

Teilweise tritt „neurologische Komorbidität“ erst im Verlauf und nach der Entlassung aus der Rehabilitation auf:

- Die Gefahr für das Auftreten epileptischer Anfälle bleibt in der Nachsorge bislang wenig berücksichtigt. Ischämische und hämorrhagische Infarkte sind die häufigste Ursache für strukturelle fokale epileptische Anfälle im Alter. Abhängig von der Schwere und der Lokalisation des Schlaganfalls beträgt das Risiko für epileptische Anfälle bis zu 7 %, mit der Gefahr einer höheren Mortalität der Betroffenen [16].
- 30 % der Schlaganfallpatienten erleben im Verlauf eine Depression. Diese gilt es zu erkennen und adäquat zu behandeln, auch mit Psychopharmaka. Problematisch ist, dass viele der neueren Antidepressiva Interaktionen mit gerinnungsaktiven Substanzen haben [28].
- Medizinische, psychologische und soziale Hilfestellungen sind nicht definiert, insbesondere wird die Tatsache der Bedeutung der Behinderung in der Folge des Schlaganfalls nicht weiter ausgearbeitet. Es gibt keine Verknüpfung von medizinischen und sozialen Leistungen im Verlauf.

Dargestellt und kritisiert wird das Fehlen von Netzwerken mit integrierter Nachsorge nach einem Schlaganfall. Es fehlt an Organisationsformen für eine ambulante Versorgung mit interprofessioneller Kooperation und Kommunikation.

In Deutschland gibt es keine Verfahren für die Einbindung der Familie in der Versorgung des Betroffenen.

### Praktikabilität von Leitlinien

Die aktuellen Leitlinien zur Nachsorge von Schlaganfällen sind teilweise umfassend formuliert und geben einzelne Behandlungsoptionen vor, liefern aber, wie bei der DEGAM-Leitlinie [8], keine konkreten Hinweise für zu leistende Nachuntersuchungen oder die aufzubauenden Nachsorgestrukturen. Für valide Eckpunkte oder Qualitätsindikatoren reicht die aktuelle Studien- und Evidenzlage nicht aus (Tab. 4).

**Table 4:** 

	Gesellschaft	AWMF-Register-Nr.
Sekundärprophylaxe ischämischer Schlaganfall und transitorische ischämische Attacke. Entwicklungsstufe: S3	Deutsche Schlaganfall-Gesellschaft (DSG) Deutsche Gesellschaft für Neurologie (DGN)	030/133
Schlaganfall-Leitlinie. Entwicklungsstufe: Entwicklungsstufe: S3	Deutsche Gesellschaft für Allgemeinmedizin und Familienmedizin e. V.	053-011 und DEGAM-Leitlinie Nr. 8
Therapie des spastischen Syndroms. Entwicklungsstufe: S2k-Leitlinie	Deutsche Gesellschaft für Neurologie (DGN)	030/078
Rehabilitation sensomotorischer Störungen. Entwicklungsstufe: S2k	Deutsche Gesellschaft für Neurologie (DGN)	030/123
Rehabilitation aphasischer Störungen nach Schlaganfall. Entwicklungsstufe: S1	Deutsche Gesellschaft für Neurologie (DGN)	030/090
Multiprofessionelle neurologische Rehabilitation. Entwicklungsstufe: S1	Deutsche Gesellschaft für Neurologie (DGN)	030/122
Hausärztliche Risikoberatung zur kardiovaskulären Prävention. Entwicklungsstufe: S3	Deutsche Gesellschaft für Allgemeinmedizin und Familienmedizin e. V.	053-024
Diagnostik, Therapie und Nachsorge der extrakraniellen Karotisstenose. Entwicklungsstufe: S3	Deutsche Gesellschaft für Gefäßchirurgie und Gefäßmedizin Deutsche Gesellschaft für Neurologie Deutsche Schlaganfallgesellschaft Deutsche Gesellschaft für Neuroradiologie	004-028

Aus Sicht des ambulant tätigen Neurologen gibt es zwar eine ausreichende Zahl von Leitlinien mit Bezug zum Schlaganfall, die nicht immer für den Routinegebrauch anwendbar sind bzw. hierfür angepasst werden sollten. Von der DGN gibt es z. B. die Leitlinie „Multiprofessionelle neurologische Rehabilitation“ [3], diese ist zu umfassend für den allgemeinen ambulanten Gebrauch. Regelmäßig überarbeitete Leitlinien der DGN liegen zu den verschiedensten Aspekten der Akut-Schlaganfall-Behandlung und zur Sekundärprävention vor.

### Patientenbedarf

Wichtig erschien allen Beteiligten die konsequente Einbeziehung von Patienten und Angehörigen bei der Nachsorge, zur Beratung über die individuellen Risiken, deren Prophylaxe und zur weiteren Behandlung.

Patientenbedürfnisse in der Schlaganfallnachsorge, die im Rahmen von „patients’ needs surveys“ untersucht werden, werden vornehmlich im englischsprachigen Ausland erfasst. Erstmals für Deutschland hat die MAS-I-Studie Patientenbedarfe erhoben [14]. Eine gute Zusammenfassung der Versorgungssituation findet sich für Deutschland bei Schwarzbach et al. Die aus Patientensicht nicht erfüllten Bedürfnisse spiegeln die von den Gruppenmitgliedern erkannten Probleme in weiten Teilen wider:

- Patienten und deren Pflegende fühlen sich nicht genug unterstützt, bspw. beim Wechsel (stationärer zur ambulanter Versorgung) zwischen verschiedenen Versorgungsektoren [2] oder bei der Beendigung von Versorgungsleistungen [2].
- Viele Patienten wünschen sich eine höhere Intensität, Häufigkeit und Dauer der Therapie [2, 23]. Hier fehlt es an Aufklärung über die Notwendigkeit.
- Lücken bestehen in Deutschland vor allem in der Versorgung mit Physiotherapie, Logopädie und Neuropsychologie [26], obwohl Konzentrationsprobleme und Probleme des Erinnerungsvermögens zu sehr häufig berichteten Problemen der Betroffenen [17] zählen.
- Der Wunsch nach einer längerfristigen Unterstützung ist mit einem erhöhten Informationsbedarf im gesamten Versorgungspfad gekoppelt. Dazu gehören Informationen zur Ätiologie, Prognose und Sekundärprävention des Schlaganfalls [2, 17, 23].
- Gerade jüngere Schlaganfallpatienten haben Bedürfnisse gemäß ihrer persönlichen Situation, die sich durch allgemeine Informationen kaum beantworten lassen. Dazu gehört der dringende Wunsch, nach der Erkrankung die Berufstätigkeit wiederaufzunehmen [2].
- Depressionen im Krankheitsverlauf des Schlaganfalls werden häufig nicht erkannt oder therapiert [20]. Dabei ist eine Depression als Folge auf den Schlaganfall assoziiert [19].
- Mehr als die Hälfte der Patienten macht sich Sorgen und Gedanken wegen möglicher Nebenwirkungen der verordneten Arzneimittel [11]. Diese werden häufig nicht von den Ärzten angesprochen oder diskutiert.
- Als wichtige „Querschnittsthemen“, die jenseits der Leistungen des SGB V für Schlaganfallpatienten und ihre Angehörigen eine wichtige Rolle spielen und damit im Rahmen der ärztlichen Versorgung adressiert werden, sind u. a. zu nennen: Umgang mit Belastungen, Organisation und Finanzierung von Hilfen und Unterstützungsmaßnahmen im Alltag, Inklusion, Partizipation [29].

## Zusammenfassung und Vorschläge der Arbeitsgruppe zur Verbesserung

In den weiteren Arbeitsgruppen der Nachsorgekommission der DSG werden Möglichkeiten und Konzepte vorgestellt, die in absehbarer Zeit umsetzbar sein sollten.

Ein wichtiges Kriterium für die weitere Nachsorge ist der sektorenübergreifende multiprofessionelle Ansatz. Neben dem Hausarzt des Patienten sollte der Neurologe führend bei der Implementierung der Nachsorge nach einem Schlaganfall sein. Dieses kann zu einem sinnvollen Einsatz der zur Verfügung stehenden Ressourcen führen, der in aktuellen Studien verfolgt wird (SANO).

Kontrovers wird die Organisation der Nachsorge in der Kommission diskutiert. Sind Kliniken mit Stroke-Units der ideale Nachsorgepunkt? Haben die Ärzte in der Klinik die Kenntnisse der ambulanten Versorgung und sind sie in die entsprechenden Versorgungsnetzwerke mit einbezogen? Haben andererseits die niedergelassenen Neurologen den aktuellen Kenntnisstand für die Nachsorge? Zu berücksichtigen sind die weiterhin streng getrennten Versorgungssysteme. Diskutiert werden muss die Entwicklung „neurovaskulärer Praxen“, bedenkenswert ist eine Zertifizierung analog zu den Stroke-Units. Die DSG könnte beispielsweise Kurrikula erstellen. Zunehmend kommen junge Neurologen in die Praxen, die während ihrer klinischen Ausbildungsphase die moderne Schlaganfallmedizin erlernt haben.

In dieser Arbeit haben wir versucht, Verbesserungspotenziale darzustellen, auf deren Wiederholung an dieser Stelle verzichtet wird. Viele der Ideen müssen durch wissenschaftliche Studien und Projekte abgesichert und evaluiert werden, vor Implementierung dieser in der Regelversorgung. Nicht zu vernachlässigen ist eine Evaluation der Kosten unterschiedlicher Programme im Vergleich zu den evaluierten Ergebnissen.
